# The Early Mobilization of Patients on Extracorporeal Membrane Oxygenation: A Systematic Review

**DOI:** 10.3390/nursrep13020066

**Published:** 2023-04-25

**Authors:** Anastasia A. Chatziefstratiou, Nikolaos V. Fotos, Konstantinos Giakoumidakis, Hero Brokalaki

**Affiliations:** 1 Department of Nursing, National and Kapodistrian University of Athens, 15771 Athens, Greece; nikfotos@nurs.uoa.gr (N.V.F.); heropan@nurs.uoa.gr (H.B.); 2 Department of Nursing, School of Health Sciences, Hellenic Mediterranean University, 71410 Heraklion, Greece; kongiakoumidakis@hmu.gr

**Keywords:** extracorporeal membrane oxygenation, rehabilitation, mobilization, ECMO, physiotherapy, prior

## Abstract

Patients on extracorporeal membrane oxygenation (ECMO) often require prolonged periods of bed rest owing to the severity of their illness. Care is also required to maintain the position and integrity of the ECMO cannula. However, they experience a range of effects due to prolonged bed rest. This systematic review examined the possible effects of the early mobilization in patients on ECMO. The database PUBMED was searched by using appropriate keywords: “rehabilitation”, “mobilization”, “ECMO” and “extracorporeal membrane oxygenation”. The selection criteria for the article search were the following: (a) studies published in the last five years, (b) descriptive studies, (c) randomized studies, (d) published in the English language and (e) studies in adults. A total of 259 studies were found, 8 of which were finally selected. Most of the studies showed that early intensive physical rehabilitation related to a decrease in in-hospital stay and a reduction in the duration of mechanical ventilation and doses of vasopressors. In addition, improvements in the functional status and rate of mortality were observed along with a reduction in health care costs. Exercise training should be a fundamental part of the management of patients on ECMO.

## 1. Introduction

Extracorporeal membrane oxygenation (ECMO) represents a specific form of extracorporeal life support that is traditionally used in patients with severe cardiorespiratory failure where conventional therapies did not have any success. The ECMO machine is connected to a patient via plastic tubes called a cannula. The tubes are placed in large veins and arteries in the legs, neck or chest. The ECMO machine pumps blood from the patient’s body to an oxygenator that adds oxygen to it and removes carbon dioxide. The ECMO machine then sends the blood back to the patient through a pump with the same force as the heart, thereby replacing its function. The membrane surface area and the blood flow determine the maximal oxygen delivery. The capacity of oxygen exchange is referred to as a rated flow which is the volume of desaturated blood (SO_2_ 75%) that the membrane can return to an SO_2_ of 95% per minute (L/min). In addition, sweep gas is either 100% oxygen or carbogen (95% O_2_ and 5% CO_2_) which is a gas flow in liters/minute via the membrane oxygenator. Sweep gas flow rates are equal to blood flow. An increase of sweep gas flow increases CO_2_ elimination. Two types of ECMO are commonly used. Namely, the venoarterial (VA) ECMO, which is connected to both a vein and an artery, and the venovenous (VV) ECMO which connected to one or more veins [1,2]. 

Most of the time, patients with ECMO are in the intensive care unit (ICU) and the duration of their hospitalization is prolonged. In addition, the specific characteristics of the ECMO circuit and its entry point on the body, usually the femoral vein, result in an extended bed rest which is reasonably related to the development of severe prolonged neuromuscular weakness [3]. Hayes et al. (2016) showed that patients on ECMO experienced impaired physical function and leg complications both in ICU and after their discharge [4]. Moreover, Todd et al. (2017) mentioned that only 3 out of 12 patients on ECMO were awake with no sedation or light sedation and 1 patient was able to gradually transition to a chair at the bedside. Therefore, most patients need long-term rehabilitation to reestablish their functional status [5]. 

In a concerted effort to prevent the potential consequences of prolonged bed rest, many studies test the contribution of physiotherapy and early mobilization among patients on ECMO. The Pulmonary Council of the International Society for Heart and Lung Transplantation prominently established that rehabilitative potential has been cited among the prerequisites for pre-lung transplant patients [6]. In addition, Abrams et al. referred to the fact that physical deconditioning and immobilization are considered strong relative contraindications to patients on ECMO [7].

However, the prior mobilization of patients on ECMO has still not yet been widely achieved. The majority of patients remain in bed and receive only passive exercises, while rehabilitation is often started little before ICU exit. For instance, Marhong et al. (2017) pointed out that only 41% of patients on ECMO attended early mobilization (<72 h following cannulation), whereas all the patients received passive range of motion exercises in bed (100%) and only 22% were ambulated [8].

Therefore, it can be said that a carefully observed gap in knowledge has been identified regarding the potential impact of comprehensive cardiac rehabilitation started early after ECMO commencement [8,9]. At the same moment, a documented significant increase in the use of ECMO worldwide has made it plain that more extensive research is needed in the field of patients on ECMO management and especially their mobilization and intensive rehabilitation [10]. The principal aim of the present systematic review is to investigate the desired effect of prior mobilization of patients on ECMO.

## 2. Materials and Methods

The electronic database PubMed was methodically searched between September 2021 and October 2021. Exclusive articles published between 1 January 2011 and 1 October 2021 were evaluated. The keywords ‘rehabilitation’, ‘mobilization’, ‘ECMO’, ‘ambulation’ and ‘extracorporeal membrane oxygenation were searched. Data were extracted and their validity was assessed by two independent reviewers and graded using the Cochrane Collaboration tool for assessing the possible risk of bias. The manuscript is fully compliant with PRISMA reposting guidelines [11]. The PROSPERO number registration is 407793.

Published studies were considered eligible if they were in English and included adult patients on ECMO and any form of a cardiac rehabilitation program, mobilization or physiotherapy.

The primary endpoint of the study is the functional capacity of patients and their muscle strength. The secondary endpoints are the length of stay both in ICU and in total in the hospital, duration of mechanical ventilation, complications due to ECMO, blood flow on ECMO, duration of ECMO and health care cost.

**Figure 1:**
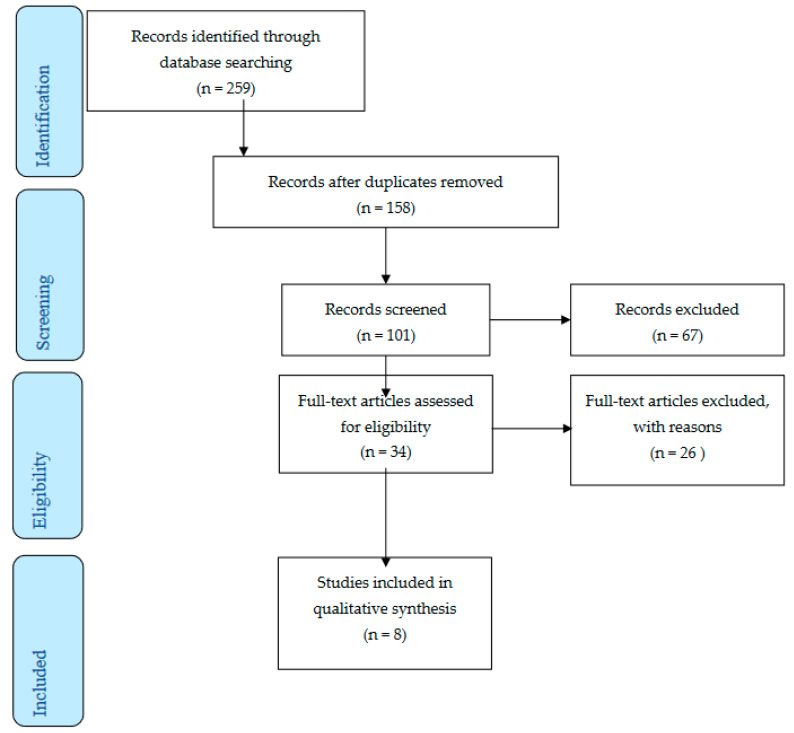
Flow chart of the selection of the studies.

Initially, 259 studies were noted and the title and abstracts of these were carefully screened. In total, 158 studies were excluded since they were found more than one time. A total of 25 were excluded after reading the title, 42 were excluded after reading the abstract and 34 were excluded after reading the full text according to inclusion and exclusion criteria. Therefore, only eight studies were included in the present systematic review. Figure 1 presents the flow chart of the selection of the studies. 

## 3. Results

In total, eight studies were included in the present systematic review. Four studies compared the potential effect of mobilization between ambulated and not-ambulated patients on ECMO, one evaluated the effect of prior mobilization and one assessed the effect of rehabilitation between patients on ECMO and without ECMO. Seven studies follow a retrospective cohort design, whereas only one is a randomized control trial. Specific details of the including studies are presented in Table 1 and Table 2.

In 2014, Abrams et al. examined the likely outcomes of early physical rehabilitation in patients on ECMO as either a bridge to transplant or a bridge to recovery due to refractory respiratory or cardiac failure [12]. The main diagnosis was cystic fibrosis, acute respiratory distress syndrome, interstitial lung disease, chronic obstructive lung disease and pulmonary arterial hypertension. In total, 35 patients participated in the study: 19 to bridge to a transplant group and 16 to bridge to a recovery group. The results showed that 75% of the patients were mechanically ventilated; however, there was no significant difference in the amount of support needed between and during exercise sessions. Regarding the functional status, the median maximum physical score was eight for the entire sample, with a mean of eight in the bridge-to-transplant group and two in the bridge to a recovery group. For the entire sample, 32% achieved a bed-level active-assisted range of motion, 6% achieved sitting in bed, 3% achieved sitting at the edge of the bed in one patient, 9% achieved standing in three patients and 51% ambulating. The average exercise sessions were 5 per patient which represents 2.8 sessions per patient each week. The median walking distance for patients who achieved ambulation was 175 feet and 2 patients managed a bedside stationary bicycle whereas one patient walked unassisted up to 2.800 feet every day. A total of 13 participants enhanced their physical function at the end of the exercise sessions, 19 maintained the same level and only 3 experienced a deterioration in their physical score. 

As for the ECMO parameters, there was no need to adjust the mean ECMO blood flow rates or sweep gas flow rates or the vasopressor doses during rehabilitation. Finally, complications associated with either the patient or ECMO circuit due to physical activity were not observed. In 2015, Ko et al. investigated the impact of early mobilization among patients on ECMO in terms of the feasibility and patient safety [13]. A total of eight patients on ECMO enrolled in the study. The intervention included a passive range of the proper motion of extremities and electrical muscle stimulation in supine, sitting in reclined bed with the head and trunk upright or on the edge of the bed, strengthening using the elastic band in a sitting position, standing out of bed, or marching in place with or without the device and walking with assistance.

A total of 62 sessions took place and half of them were conducted via a passive range of motion. In total, 27.4% was performed during sitting in bed with the head and trunk upright or on the edge of the bed, 3.2% were for strengthening using the elastic band in a sitting position and 18% for patients who were standing out of bed or marching in place with or without standing device. Whereas, only 2% was conducted for patients who were walking with assistance. The blood flow rate of ECMO was higher during rehabilitation sessions than before (*p* = 0.013). On the contrary, a fundamental difference in the sweep gas flow rate of ECMO before physical activity compared to during the sessions was not observed (*p* = 0.321). Finally, only 5% of the sessions were stopped due to side effects. More specifically, one episode of tachycardia (132 beats/minute) and two episodes of tachypnea (46 and 47 per minute), respectively, were observed. 

In 2016, Bain et al. examined the possible difference in hospital cost between ambulated patients on ECMO and non-ambulated patients on ECMO [14]. A total of nine patients on ECMO as a bridge to lung transplantation enrolled in the study. Four patients typically received the usual care whereas the remaining five underwent active physical rehabilitation including ambulation. The findings indicated significant clinical differences between the two groups. For instance, the pre-transplant ICU stay was 12 days (4–41) for the usual care group and 20 days (17–30) for the rehabilitation group (*p* = 0.32), whereas the post-transplant ICU stay was 45 (34–56) and 8 (6–22) (*p* = 0.01), respectively. Moreover, the pre-transplant mechanical ventilation duration was 1 day (1–5) for the usual care group and 12 days (5–15) for the rehabilitation group (*p* = 0.02) and the post-transplant mechanical ventilation duration was 29.5 (22–54) and 2 (1–5) (*p* = 0.01), respectively.

In addition, patients in the rehabilitation group were characterized by both shorter post-ICU stays and total hospital stays compared to the usual care group of 11 days (7–25) vs. 34 days (11–63) (*p* = 0.06) and 50 (31–63) vs. 94 (51–151), respectively. On the contrary, patients in the training group were on ECMO for a longer period of time than the usual care group, 9 days (5–14) vs. 1.5 days (1–9) (*p* = 0.06). Regarding total cost, the health care cost was higher for the usual care group than the training group, $300,307 ($274,262–394,913) vs. $244,508 ($219,972–268,914) (*p* = 0.02). 

Wells et al. (2017) conducted a retrospective cohort study with the aim to estimate the patient safety and feasibility of rehabilitation sessions among patients on ECMO as a bridge to transplant [9]. Two hundred fifty-four patients on ECMO participated in the study who subdivided into two groups, the intervention group (*n* = 167) and the usual care group (*n* = 87). A total of 268 exercise sessions were provided with 51 of these occurring at the edge of the bed or in a chair, 170 interventions of bed mobility (rolling, supine to sit transfer training and bridging activities) and 106 sitting activities (sit to standing transfers and functional strengthening using sit to stand from the bed or chair). In total, 39 transfer interventions (patient completing pivot or taking small steps from the bed or chair with a purpose to transfer to another surface), 98 standing activities (standing balance and tolerance, strengthening, pre-ambulation activities such as weight shifting, marching and stepping in place) and 37 ambulation interventions (gait training, gait speed and ambulation tolerance) were recorded. 

The results indicated that the mean duration of support time on ECMO was 393 h for the intervention group which was slightly longer than the average duration of support for the entire group. In addition, only two minor episodes of nonsustained ventricular tachycardia for one patient during standing activities were observed and three minor events (<0.5%) required the termination of the activity. However, no major events occurred during the 607 exercise sessions. The range of the walking distance was 30 to 1000 feet. 

In addition, the majority of the intervention group (65.3%) survived hospital discharge, 23.9% of them were discharged home, 68.8% were discharged to a rehabilitation facility and 3.7% were discharged to skilled nursing facilities. On the other hand, only 22.2% of patients who received exercise sessions only after decannulation were discharged home, 63.9% were discharged to a rehabilitation facility and 11.1% went to skilled nursing facilities. 

During the same period of time, Munshi et al. (2017) described the outcomes of exercise training in the management of patients with acute respiratory distress syndrome (ARDS) on ECMO [15]. One hundred seven patients on ECMO as a bridge to recovery enrolled in the study. The sample was subdivided into two groups: the exercise group (84%) and the usual care group (16%). The findings of the study showed that the duration of mechanical ventilation was longer in the exercise group, 72 h vs. 28 in the usual care group (*p* = 0.12), whereas the level of severity was higher in the usual care group: APACHE II pre-ECMO was 28 (±9) vs. 27 (±8) (*p* = 0.63). In addition, patients in the exercise group achieved an improvement on ECMO parameters since the ECMO flow was lower in this group 4.4 vs. the usual care group 4.6 (*p* = 0.72) as well as the ECMO sweep 4.0 vs. 4.5 (*p* = 0.39). As for mortality, the rate of mortality was lower both in the intensive care unit and in the hospital since one death in the exercise group was registered vs. seven deaths in the usual care group (*p* = 0.006). On the contrary, the duration of ECMO was longer in the training group 13 days vs. 8 in the usual care group (*p* < 0.001). 

In 2018, Hayes et al. examined the effect of exercise training in patients on ECMO before and after lung transplantation [16]. Moreover, researchers compared the possible outcomes between patients after lung transplantation not requiring ECMO. A total of 42 patients after lung transplantation took part in the study of whom 17 were on ECMO. The training before lung transplantation included outpatient supervised exercise training classes 2–3 times a week based on established pulmonary rehabilitation guidelines. On the other side, the program after lung transplantation for patients requiring ECMO was typically begun in the ICU as early as the first postoperative day with the goal of achieving the highest level of mobility. In addition, while patients were on ECMO the rehabilitation program included resistance and motion exercises for the upper and lower limbs, progressing to sitting, standing and, ultimately, ambulation as medical stability allowed. After patients were discharged from ICU, patients after lung transplantation enrolled in 12 weeks of supervised, gym-based, aerobic and strengthening exercises for 1 h 3 times a week. 

Patients’ mobility was assessed using the ICU mobility scale (IMS) and functional capacity via a 6-min walking test (6MWT) whereas muscle strength was estimated using the Medical Research Council sum score (MRC). 

The results indicated individuals after lung transplantation requiring ECMO were characterized by prolonged mechanical ventilation, longer ICU and hospital stay compared with those not requiring ECMO. In addition, the ECMO lung transplant group achieved a lower mobility level (IMS median 6) vs. the non-ECMO group (IMS median 7). Moreover, patients in the ECMO group needed more time to reach mobility milestones compared to the non-ECMO group and ECMO lung transplant patients achieved less distance in 6MWT at hospital discharge (mean difference: 99 m, 95% CI: 33 to 165, *p* < 0.004). However, according to multiple regression analyses, the duration of stay in the ICU was the only predictor factor of lower distance based on 6MWT. 

Finally, both groups experienced an improvement in 6MWT at three months without any significant difference between the two groups. As for muscle strength, ECMO patients’ MRC strength score was 48/60 which indicated muscle weakness. However, the muscle strength enhanced after hospital discharge without achieving the normal levels (mean improvement MRC 11, 95% CI 7–15, *p* < 0.001). Regarding ECMO patients experiencing a leg complication, they achieved the completion of a 12-week post-transplant rehabilitation program.

One year after, Bonizzoli et al. (2019) aimed to examine the effect of early physiotherapy (first session within the first week from ECMO start) in the management of patients with Acute Respiratory Disease Syndrome (ARDS) [17]. A total of 101 patients on venovenous ECMO enrolled in the study. The findings showed that early physiotherapy was related to a lower duration of ECMO support (*p* = 0.03), mechanical ventilation (*p* = 0.001) and duration of stay in the ICU (*p* = 0.001). However, a statistically significant difference in the mortality rate was not observed. At linear regression analysis, the time from ECMO start to first of the physiotherapy session was associated with the length of stay (r^2^ = 0.48, *p* < 0.001). Finally, at the beginning of the physiotherapy, 65.3% of individuals were able to perform exercises only in bed; however, at discharge, 37.3% were able to actively participate in physiotherapy. 

In 2021, Hayes et al. conducted a pilot study with the aim of assessing the effect of an intensive cardiac rehabilitation program in patients on ECMO due to ARDS, pots lung or heart transplantation primary graft failure, cardiac arrest, cardiac failure infraction and pulmonary hypertension after lung transplantation [18]. A total of, 15 patients were enrolled who were sub-divided into the cardiac rehabilitation group and the group receiving the usual care. The exercise was conducted for approximately one hour per day with a minimum time of 20 min if passive exercise was performed and 30 min if active exercise was undertaken. The training could be continuous or intermittent throughout the day, whereas the intensity was targeted at a perceived exertion level of three to five on the Borg scale. 

The results indicated that the highest level of mobilization is positively associated with a higher respiratory rate and maximum tidal volume during physiotherapy. In addition, it reduced the dosage of noradrenaline and the ECMO fresh gas flow and less sedation was needed, whereas the exercise duration was longer for the cardiac rehabilitation group than the usual care group. More specifically, the mean duration of exercise in the rehabilitation group was 28.7 min vs. 4.2 in the usual care group (*p* < 0.0001). Three out of seven patients in the rehabilitation group mobilized out of bed whilst on ECMO vs. none in the usual care group. Finally, patients in the rehabilitation group achieved standing sooner than patients in the usual care group.

## 4. Discussion

The present systematic review was carried out with the aim to investigate the notable contribution of mobilization in the management of patients on ECMO. The review of the electronic database identified eight studies that fulfilled the inclusion and exclusion criteria of the present systematic review. 

Some studies have shown that exercise interventions might reduce the length of ICU stays and decrease the ventilation duration [9,14,15]. Bain et al. (2014) found out that ICU stay duration and mechanical ventilation duration were longer in patients on ECMO participating in exercise training before transplantation, whereas individuals in the exercise group were characterized by a shorter duration of ICU stay and ventilation after transplantation [14]. Likewise, Munshi et al. (2017) showed that the duration of mechanical ventilation was longer in the exercise group 72 h vs. 28 in the usual care group (*p* = 0.12), which was associated with the level of severity [15]. The results of the study by Hayes et al. (2018) positively reinforced the previous finding since they concluded that patients after lung transplantation needed ECMO support characterized by a longer duration of ICU stay and mechanical ventilation [16]. The fact is associated with the severity of the patient’s clinical status since their hemodynamic instability and respiratory failure required patient support with ECMO. 

On the contrary, the results are controversial regarding the need to adjust ECMO parameters such as blood flow rates or sweep gas during exercise training [12,13]. Abrams et al. (2014) mentioned that there was no need for an increase in the blood flow rate or swap gas flow rate [12], whereas Ko et al. (2015) found a need for an increase in the blood flow rate without any change in the sweep gas flow rate [13]. However, Munish et al. (2017) proved that patients in the exercise group achieved a marked improvement on ECMO parameters since they achieved lower ECMO flow and ECMO sweep [15]. Moreover, Hayes et al. (2018) indicated that the highest level of mobilization is associated with a higher respiratory rate and maximum tidal volume during physiotherapy and a gradual reduction in the dosage of noradrenaline, the ECMO fresh gas flow and the level of sedation needed [16]. The change in ECMO parameters may be associated with the type and the intensity of exercise as well as the patients’ clinical characteristics, therefore more research is needed in this field.

As for physical function, only two studies identified negotiating the potential effect of ECMO support on patients’ physical function and the role of exercise training during ECMO support. Abrams et al. (2014) mentioned that 13 participants on ECMO who were mobilized experienced a considerable improvement in their physical function at the end of the exercise sessions, 19 maintained the same level and only 3 experienced a deterioration in their physical score [12]. However, Hayes et al. (2018) showed that patients after lung transplants who supported ECMO achieved lower mobility levels compared to patients after lung transplants who were not supported by ECMO. In addition, patients in the ECMO group needed more time to reach mobility milestones compared to the nonECMO group and ECMO lung transplant patients achieved less distance in 6MWT and lower muscle strength at hospital discharge [16]. 

It is imperative to mention the results of the Bonizzoli et al. (2017) study since they have given prominence to the time of the beginning of exercise sessions since ECMO implantation. More specifically, researchers indicated that prior mobilization was related to the lower duration of ECMO support and in-hospital stay [17]. 

The results of the studies listed in this systematic review demonstrate that multimodal exercise training approaches used in the rehabilitation of patients on ECMO support are considered safe because of the absence of severe events and the small number of mild adverse events [9,12,13]. In the published study by Ko et al. (2015), only 5% of the exercise sessions were stopped due to side effects since one episode of tachycardia (132 beats/minute) and two episodes of tachypnea (46 and 47 per minute) was observed [13]. Likewise, Wells et al. (2018) referred to only two minor episodes of nonsustained ventricular tachycardia which undoubtedly required the termination of the activity. However, no major events occurred during the 607 exercise sessions [9]. The above results amplify the patient safety on ECMO ambulation due to the advances in developed ECMO technology which allow for the mobilization of patients [18,19,20,21,22].

As for mortality, Munshi et al. (2017) showed that the rate of mortality was lower both in the intensive care unit and in the hospital in the exercise group since one was registered one death in the training group vs. seven deaths in the usual care group (*p* = 0.006) [15]. On the contrary, Bonizzoli et al. (2019) support that no statistically significant difference was observed in the mortality rate between the above two groups [17] and the rate of fatal outcomes, although the probability of reducing mortality has not been confirmed. Finally, only one published study examined the cost-effectiveness of exercise training among patients on ECMO. In particular, the total health care cost was higher for the usual care group than the training group: $300,307 ($274,262–394,913) vs. $244,508 ($219,972–268,914) (*p* = 0.02) [14].

It is important to refer to some limitations of the included studies. First of all, none of the studies enrolled patients on both VV ECMO and VA ECMO and compared the results between these groups [15,17]. Therefore, they did not provide information regarding possible complications or appropriate vascular access. Moreover, half of the studies included patients with different health conditions such as ARDS, after lung transplantation, acute heart failure, chronic obstructive pulmonary disease, pulmonary hypertension and cardiac arrest [13,15,17,18]. However, none of these studies examined the findings within the groups in order to test the safety and the contribution of ECMO in each patient group. 

Finally, all studies are characterized by a lack of reproducibility since they did not provide much information regarding the rehabilitation or mobilization program. More specifically, most of them referred to the type of exercise such as bed-level active-assisted range of motion, sitting in bed and sitting at the edge of the bed in one patient; however, they did not mentioned the duration and the frequency of each exercise. 

## 5. Conclusions

Preventing the deleterious effects of prolonged bed rest has many benefits, including the reduction of mechanical ventilation, in-hospital stay and vasopressors needed together with improved functional capacity. All the above result in a decrease in the rate of mortality and total health care cost. However, the number of these outcomes was not sufficiently large to provide an adequate level of evidence.

## Figures and Tables

**Table 1:** Main characteristics of the studies.

Authors/Year	Country	Type	*N*	Intervention	Results
Abrams et al. [12]/2014	USA	Retrospective cohort study	35	Group 1: patients on ECMO as a bridge to transplant (*N* = 19) Group 2: patients on ECMO as a bridge to recovery (*Ν* = 16)	A total of 13 participants had enhanced their physical function at the end of the exercise sessions. A total of 19 maintained the same level and only three experienced deteriorations in their physical score. Νo need to change the mean ECMO blood flow rates, sweep gas flow rates or vasopressor doses was identified during rehabilitation. There were no complications associated with either patients or ECMO circuit due to physical activity.
Ko et al. [13]/2015	South Korea	Retrospective single-center study	8	One group on ECMO	The blood flow rate was higher during a rehabilitation session. No difference in the sweep gas flow rate of ECMO. Only 5% of the sessions were stopped due to side effects.
Bain et al. [14]/2016	USA	Single-center retrospective cohort study	9	Group 1: exercise training (*Ν* = 5) Group 2: usual care (*Ν* = 4)	Longer duration of mechanical ventilation, ICU and total hospital stay for group 2. Lower health care cost for group 1.
Wells et al. [9]/2017	USA	Retrospective cohort study	254	Group 1: exercise training group (*Ν* = 167) Group 2: usual care (*Ν* = 87)	Longer duration on ECMO in group 1, 393 h for group 1 vs. 306 h for group 2. More patients from group 1 discharged at home and rehabilitation services. Two episodes of nonsustained ventricular tachycardia and no major events occurred.
Munshi et al. [15]/2017	Canada	Retrospective cohort study	107	Group 1: exercise training (*N* = 50) Group 2: usual care (*N* = 11)	Longer duration of mechanical ventilation in the exercise group 72 h vs. 28 in the usual care group (*p* = 0.12). Improvement on ECMO parameters for group 1. The rate of mortality was lower both in the intensive care unit and in the hospital in group 1. The duration of ECMO was longer in the training group (*p* < 0.001).
Hayes et al. [16]/2018	Australia	Retrospective single-center study	42	Group 1: patients on ECMO (*N* = 17) Group 2: patients without ECMO (*N* = 25)	Longer duration of mechanical ventilation, ICU stay and hospital stay for group 1. Lower mobility level and more time needed to reach mobility milestones in group 1. Muscle strength entailed weakness in group 1 without achieving normal levels after discharge.
Bonizzoli et al. [17]/2019	Italy	Retrospective observational study	101	Group 1: physiotherapy within the 1st week from ECMO (*N* = 33) Group 2: physiotherapy after the 1st week from ECMO (*N* = 68)	Lower duration of ECMO support, mechanical ventilation and duration of stay in group 2. Nonsignificant difference in mortality.
Hayes et al. [18]/2021	Australia	A multicenter randomized controlled study	15	Group 1: cardiac rehabilitation moderate intense (*N* = 7) Group 2: usual care (*N* = 8)	Higher respiratory rate and maximum tidal volume during physiotherapy. Reduction of noradrenaline and ECMO fresh gas flow. Longer exercise duration for the cardiac rehabilitation group. Three out of seven patients in the rehabilitation group were mobilized out of bed whilst on ECMO vs. none in the usual care group. Patients in the rehabilitation group achieved standing sooner than patients in the usual care group.

**Table 2:** Results of the studies.

Authors/Year	Diagnosis	Group	Results
Abrams et al. [12]/2014	Refractory respiratory or cardiac failure	Bridge to recovery *N* = 19	ECMO blood flow rate pre-PT (LPM, mean ± SD): 3.00 ± 0.99 ECMO blood flow rate during PT (LPM, mean ± SD): 2.92 ± 1.09 ECMO sweep gas flow rate pre-PT (LPM, mean ± SD): 2.39 ± 1.77 ECMO sweep gas flow rate during PT (LPM, mean ± SD): 2.35 ± 1.78 Dose of norepinephrine (mcg/min, median, IQR): 1.3 (0.5 to 2) Dose of vasopressin (units/min): 0.04 Maximum distance ambulated (ft, median, IQR): 170 (55 to 525)
		Bridge to transplant *Ν* = 16	ECMO blood flow rate pre-PT (LPM, mean ± SD): 2.99 ± 0.81 ECMO blood flow rate during PT (LPM, mean ± SD): 3.02 ± 0.82 ECMO sweep gas flow rate pre-PT (LPM, mean ± SD): 3.45 ± 1.71 ECMO sweep gas flow rate during PT (LPM, mean ± SD): 3.46 ± 1.71 Dose of norepinephrine (mcg/min, median, IQR): 3.5 (1.3 to 5) Dose of vasopressin (units/min): 0.04 Maximum distance ambulated (ft, median, IQR): 195 (60 to 398)
Ko et al. [13]/2015	Unknown	Bridge to transplantation *Ν* = 8	Blood flow before PT: 2.93 ± 0.93 During PT: 3.02 ± 0.90 Sweep gas flow before PT: 4.89 ± 1.78 During PT: 4.90 ± 1.78
Bain et al. [14]/2016	Unknown	Bridge to transplantation Non-rehabilitation group *N* = 4	Pre-transplant: $52,124 ($23,824–69,929) Post-transplant ICU: $143,407 ($112,199–168,993) Post-ICU through discharge: $143,407 ($112,199–168,993) Total hospital: $273,291 ($237,299–374,175) Total: $300,307 ($274,262–394,913) Pre-transplant ICU stay: 12 (4–41) Pre-transplant mechanical ventilation duration: 1 (1–5) Post-transplant mechanical ventilation duration: 29.5 (22–54) Post-transplant ICU stay: 45 (34–56) Post-ICU to discharge stay: 34 (11–63) Total hospital stay: 94 (51–151) ECMO support: 1.5 (1–9)
		Bridge to transplantation Rehabilitation group *N* = 5	Pre-transplant: $98,460 ($38,589–122,111) Post-transplant ICU: $43,929 ($23,611–64,126) Post-ICU through discharge: $15,544 ($11,499–43,870) Total hospital: $209,590 ($166,767–264,536) Total: $244,508 ($219,972–268,914) Pre-transplant ICU stay: 20 (17–30) Pre-transplant mechanical ventilation duration: 12 (5–15) Post-transplant mechanical ventilation duration: 2 (1–5) Post-transplant ICU stay: 8 (6–22) Post-ICU to discharge stay: 11 (7–25) Total hospital stay: 50 (31–63) ECMO support: 9 (5–14)
Wells et al. [9]/2017	Unknown	Bridge to transplantation Total *N* = 254	Discharge outcomes, *N* (%) Home: 34 (13.38) Rehabilitation facility: 96 (37.79) Skilled nursing: 10 (2.82) Acute care facility: 5 (1.96)
		Bridge to transplantation Physical therapy group *N* = 167	Discharge outcomes, *N* (%) Home: 26 (15.56) Rehabilitation facility: 75 (44.91) Skilled nursing: 4 (2.39) Acute care facility: 4 (2.39)
Munshi et al. [15]/2017	ARDS	*N* = 61	Days on ECMO: 12 (9–19) Duration of mechanical ventilation: 21 (18–34) ICU mortality: 18 (30) In hospital mortality: 18 (30) Complications on ECMO Barotrauma: 4 (7) Limb ischemia: 1 (2) Intracerebral hemorrhage: 1 (2) Heparin induced thrombocytopenia: 4 (7) Air embolism: 1 (2)
Hayes et al. [16]/2018	Cystic fibrosis/bronchiectasis, COPD, asthma, and obliterative bronchiolitis, Pulmonary hypertension, Pulmonary fibrosis, Re-transplant	ΕCMO *Ν* = 42	Physical function IMS ICU at discharge: 6 (5–7) IMS at hospital discharge: 10 (9–10) 6MWD at hospital discharge, m: 285 ± 112 6MWD at 3 months, m: 541 ± 133 Discharge destination, *N* (%) Home: 12 (85.7) In-patient rehabilitation: 2 (14.3)
		Non-ECMO *N* = 28	Physical function IMS ICU at discharge: 7 (6–8) IMS at hospital discharge: 10 (10–10) 6MWD at hospital discharge, m: 384 ± 93 6MWD at 3 months, m: 584 ± 67 Discharge destination, *N* (%) Home: 28 (100)) In-patient rehabilitation: 0 (0)
Bonizzoli et al. [17]/2019	ARDS	Within the first week mobilization *N* = 33	ECMO (days) (median, IQR): 7 (2.5–13.5) MV (days) (median, IQR): 11 (5–17.75) LOS (days) (median, IQR): 12 (7.25–21) In-ICU mortality: 12
		After the first week *N* = 68	ECMO (days) (median, IQR): 11 (8.75–22) MV (days) (median, IQR): 23 (13.75–33.25) LOS (days) (median, IQR): 25 (18.75–36.25) In-ICU mortality:14
Hayes et al. [18]/2021	ARDS, pots lung or heart transplantation primary graft failure, cardiac arrest, cardiac failure- infraction and pulmonary hypertension after lung transplantation	Mobilization group *N* = 7	Hospital outcomes In-hospital mortality: 3 (42.9) ECMO duration (days): 8.1 ± 4.9 ECMO duration for survivors: 10.5 ± 5.5 Ventilation (days): 6.2 ± 2.5 Ventilation for survivors: 7.3 ± 2.8 LOS in the ICU (days): 12.9 (7.2 ± 16.7) LOS in the ICU for survivors: 16.7 (14.6 ± 21.6) LOS in the hospital for survivors: 41.9 (34.3 ± 56.4) Mobility milestones Time to first SOOB (days): 12.6 ± 6.6 Time to first stand (days): 5.5 ± 4.5 Time to first walk (days): 16.1 (11.5 ± 21.0) Discharge destination of survivors Home: 4 (100) Inpatient rehabilitation: 0 (0) Transfer to the local acute hospital: 0 (0)
		Usual care group *Ν* = 8	Hospital outcomes In-hospital mortality: 1 (12.5) ECMO duration (days): 10.9 ± 5.5 ECMO duration for survivors: 11.5 ± 5.7 Ventilation (days): 9.2 ± 3.8 Ventilation for survivors: 9.4 ± 4.1 LOS in the ICU (days): 21.4 (15.5 ± 38.5) LOS in the ICU for survivors: 22.2 (16.2 ± 38.5) LOS in the hospital for survivors: 34.4 (29.3 ± 87.2) Mobility milestones Time to first SOOB (days): 12.5 ± 7.7 Time to first stand (days): 20.8 ± 12.3 Time to first walk (days): 21.9 (16.5±52.4) Discharge destination of survivors Home: 3 (43) Inpatient rehabilitation: 3 (43) Transfer to the local acute hospital: 1 (14)

ARDS: Acute Respiratory Distress Syndrome; PT: Post transplantation; LPM: liters per minute; IQR: interquartile range, ΡΤ: Physical Therapy; ICU: Intensive Care Unit, MV: mechanical ventilation, LOS: length of stay, SOOB: sit out of bed.

## Data Availability

Not applicable.
